# Evolution of Hepatitis B Virus in a Chronic HBV-Infected Patient over 2 Years

**DOI:** 10.1155/2011/939148

**Published:** 2011-07-12

**Authors:** Tao Shen, Xin-Min Yan, Jin-Ping Zhang, Jin-Li Wang, Rong-Xia Zuo, Li Li, Lin-Pin Wang

**Affiliations:** Institute of Basic Medicine of the First People's Hospital of Yunnan Province, Center of Clinical Molecular Biology of Yunnan Province, Kunhua Affiliated Hospital of Kunming Medical College, 157 Jinbi Road, Yunnan Province, Kunming 650032, China

## Abstract

Mutations in full-length HBV isolates obtained from a chronic HBV-infected patient were evaluated at three time points: 1 day, 6 months, and 31 months. While 5 nucleotides variation, and an 18 bp deletion of preS1 have been kept in during at least the first two years, C339T mutation occurring in the hydrophilic region of HBsAg and T770C that caused polymerase V560A substitution were the new point mutations found existing in sequenced clones of the 3rd time point. Internal deletion of coding region obviously appeared in the 3rd time point. The splicers included two new 5′-splice donors and three new 3′-splice acceptors besides the reported donors and acceptors and may have produced presumptive HBV-spliced proteins or truncated preS proteins. ALT, HBeAg and viral DNA load varied during the follow-up years. These data demonstrated the diversity of genomes in HBV-infected patient during evolution. Combined with clinical data, the HBV variants discovered in this patient may contribute to viral persistence of infection or liver pathogenesis.

## 1. Introduction

Chronic hepatitis B virus (HBV) infection is a significant public health threat. Approximately 3-4 billion people worldwide have been infected with HBV. The natural course of chronic HBV infection is variable, ranging from an inactive HBsAg carrier state to a progressive chronic hepatitis which may eventually lead to cirrhosis and hepatocellular carcinoma (HCC). Previous research has shown that patients with persistent HBeAg-positive state and high DNA viral load (>10^6^ copy/mL) are at higher risk of HCC than the HBeAg negative individuals with low DNA viral load [1, 2]. In order to have a better understanding of this phenomenon, we obtained HBsAg-positive serum samples from Clinical Laboratory Center of the First People's Hospital of Yunnan Province, Kunming, Yunnan, China, from February to August, 2005. We also followed up a chronically HBV-infected patient who was HBeAg positive with high HBV DNA load (≥10^7^ copy/mL) for almost 3 years and studied the evolution of her HBV strains. By sequencing and phylogenetic analysis, we found mutations occurred within the genomes of HBV through time-course evolution. Combined with clinical data, whether and how these HBV variants might contribute to viral persistence of infection or liver pathogenesis still needs to be further studied.

## 2. Materials and Methods

### 2.1. Subject and Serum Samples

A 31-year-old female with chronic HBV infection was followed up at the First People's Hospital of Yunnan Province, Kunming, Yunnan, China. The subject was admitted in the hospital because of allergic purpura in May, 2005 and was diagnosed with chronic infection based on the fact of having been HBsAg positive for at least 7 years. Her serum samples were collected at three time points after admission: 602 (1 day), 6022 (6 months), and 6023 (two years and seven moths) and stored at −80°C until time of analysis. Serological markers of HBV, ALT, AST, BIL, ALB, and GLO were analyzed at each time point. The patient was seronegative for antibodies to HAV, HCV, HEV, and HIV and did not have a history of alcohol abuse, drug abuse, or hepatotoxin exposure. No antiviral treatment or immunomodulators were given before or during the study period.

### 2.2. Amplification and Sequencing of Full-Length HBV Genome

HBV DNA was extracted from serum by proteinase K digestion, followed by phenol/chloroform extraction. Viral DNA load was determined using HBV DNA PCR-Fluorescence Quantitation kit (Kehua Bio-Tech. Co. Ltd., China). The complete HBV genome was amplified by PCR described by Günther et al. [3]. The primers used were forward primer: 5′ctttttcacctctgcctaatca3′; reverse primer: 5′agaggtgaaaaagttgcatggt3′. Amplification was performed in a 96-well cycler (Bio-RAD, USA), and 25 *μ*L PCR mixture containing 2.5 mM MgCl_2_, 200 *μ*M dNTP, 400 nM of each primer, and 2 U of Ex Taq polymerase (Takara Bio-Tech. Co. Ltd.) was used. The PCR reaction was performed using the following cycles: 94°C predenature for 5 min, 30 cycles of 94°C for 1 min, 56°C for 1 min, 72°C for 2 min; 72°C for 10 min as a final extension step. Then full-length amplicons were purified using a gel extraction kit (HuaShun Bio-Engineering Co. LTD., Shanghai, China). The PCR products were purified and cloned in vector pMD18-T (TaKaRa Bio-Tech. Co. Ltd.) using standard cloning techniques. White colonies were picked, correct recombinants were confirmed by PCR, and the double-restriction endonuclease digestion with EcoRI and Hind III. DNA sequencing analysis of the correct recombinants was performed with BigDye Terminator v3.1 and 3130 Genetic Analyzer (Applied Biosystems) (7 pairs of sequencing primers can be obtained on request). For each time point, 8-9 recombinants were randomly selected and sequenced.

### 2.3. Sequence Analysis

Fourteen HBV complete genomes available in GenBank (genotypes A, B and E to H) were applied to phylogenetic analysis. Additionally, 50 published sequences of genotype C were also used in this study for alignment analysis (Genbank accession numbers can be obtained upon request). The whole genome sequences were assembled by BioEdit sequence alignment editor software, version 7.0.5.2 [4]. Alignment was performed using Clustal X software, version 1.83 [5]. Phylogenetic and molecular evolutionary analysis of nucleotide differences within and between the isolate sequences were carried out by MEGA program, version 3.1 [6]. Genetic distance was estimated using the Kimura two-parameter algorithm. The phylogenetic trees were constructed by the neighbor-joining method. Bootstrap re-sampling and reconstruction were carried out for 1,000 times to confirm the reliability of phylogenetic trees.

## 3. Results

### 3.1. Clinic Data and HBV DNA Load(s) in Serum

As showed in Table 1, HBsAg and HBeAg were positive at all three time points and titers changed during the course of analysis. ALT levels showed a tendency of elevation, and at the 3rd time point the level was more than 2 times of the upper limit of normal. While no quantification could be obtained from the 1st time point due to the lack of serum, viral DNA load was found to decrease from 10^8^ to 10^7^ copy/mL at the last two time points.

### 3.2. Phylogenetic Analysis

26 sequences were analyzed (GenBank accession number: DQ377159-377165, EU306713-306714, EU306722–306729, EU439005, EU439008–439014 and EU439025). The divergence of these 26 sequences was found to be 0–1.4%. When compared to the 64 complete HBV genome sequences published in the GenBank, all 26 sequences were determined to be genotype C and C2 subgroup with a bootstrap value of 100% (data not shown). The serotype was classified as adrq+.

### 3.3. Difference in Nucleotide(s)

**Figure 1:**
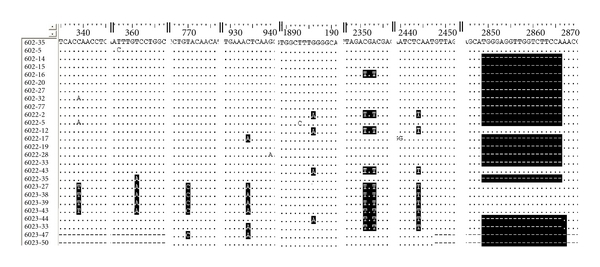
Partial nucleotide sequences of all the 26 clones obtained from three time points. Black bars indicate the major mutation during this period of time. The variants include nucleotide substitution and deletion. 602 (1st time point, 1 day); 6022 (2nd time point, 6 months); 6023 (3rd time point, 31 months).

**Figure 2:**
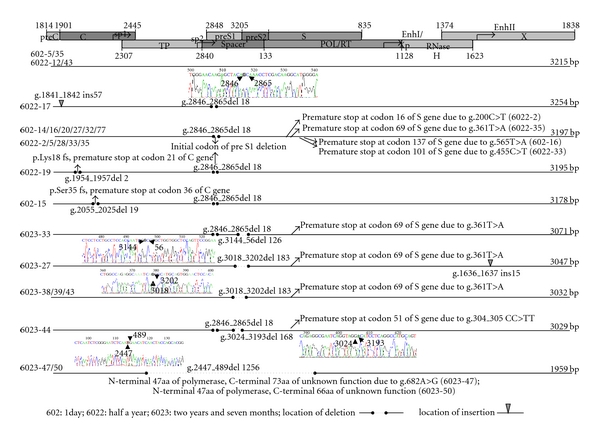
Full-length nucleotide sequences of all the 26 clones obtained from three time points. 602 (1st time point, 1 day); 6022 (2nd time point, 6 months); 6023 (3rd time point, 31 months). The length of these clones varied from 3254 bp to 1959 bp, which were caused by the insertion and deletion. Premature stop codons of S gene (g.200C>T, g.565T>A, g.361T>A, g.455C>T, and g.304_305CC>TT) are also shown.

When compared with the 50 known and complete sequences of genotype C, we found that there were 13 specific nucleotides in all sequenced clones of this patient (data not shown). Further analysis of the nucleotide sequence at all three time points indicated that nucleotides T361A, C934A, C2351T/A2353T, and C2444T were the mutation been kept in for at least two years' evolution. These mutations caused nonsense mutation of preS1 and preCore/Core gene (T361A and C2444T, resp.), missense mutation of precore/core and pol gene (C2351T/A2353T and C934A, resp.). Nucleotides C339T and T770C were the new and major mutation at the 3rd time point, and these mutations caused HBsAg P62L and polymerase (Pol) V560A mutation, respectively (Figure 1). Premature stop codons of S gene (g.200C>T, g.565T>A, g.361T>A, g.455C>T, and g.304_305CC>TT) increased during the course(1/9 versus 3/9 versus 6/8) (Figure 2). A1762T/G1764A double mutation existed in all clones, and G1896A existed in 4 clones obtained from all time points.

### 3.4. Defective Genomes during the HBV Evolution

Performing alignment showed that over the 2-year period, deletions across overlapping region of polymerase/preS1 (g.2447_489del 1256) or preS1/preS2 (g.3018_3202del 183, and g.3144_56del 126), and pre-S1 initiation codon mutations (g.2846_2865del 18) with or without internal deletions mutations (g.3024_3193del 168) were prevalent (22/26, in 85% of the clones) (Figure 2). All the defective genomes shared sequences from nucleotide position 489 to position 2447, and common to all genomes is the presence of the complete X gene and the complete C ORF. Four paires of donor/acceptor were found in the spliced genomes, besides the frequently reported donor sites 2447 and 3018 and the acceptor sites 489, two new 5′-splice donors (nt3144 and 3024) and three new 3′-splice acceptors (nt56, 3193 and 3202) were detected at the 3rd time.

## 4. Discussion

Up until now, details of the mechanism accounted for the HBV evolution remained unknown. In this study, we followed up a chronically HBV-infected patient and discovered that complex and diverse quasispecies existed in this patient for over two-year disease evolution. Based on the alignment and phylogenetic analyses, the divergence and the 13 specific nucleotides of the 26 clones, as well as the phylogenic analysis it is indicated that all sequences were directly descent from the common ancestor. Thus, these results reflect the real evolution process of homogeneous HBV strains in this patient.

### 4.1. Significance of Nonsense Mutations of PreS1/S2/S Gene

In the process of evolution, some mutations in this patient were kept in for at least 2 years, and other new mutations emerged at the 3rd time point. These mutations resulted in amino acids substitutions and some caused premature stop codon of the coding regions. Among these mutants, the T361A nonsense mutation in the small S region, which resulted in a 69aa truncated HBsAg lacking the entire “a” determinant region (aa124–147), was first detected at the 2nd time point in one clone. At the 3rd time point, however, half of the sequenced clones were found to have this mutation. This mutant has been reported in the occult HBV-infected patients with seronegative for HBsAg, and in one case HBV DNA was absent from the serum after the two-year [7, 8]. In our case, after two-year followup, the HBsAg was still at a high level and could be detected by routine immunoassay-based diagnosis. In addition, HBV DNA was at a high load despite a slightly decreased level (2.4 × 10^8^ versus 2.5 × 10^7^ copy/mL), and the ALT level showed an elevating trend over the study period. In the meanwhile, new point mutations such as C339T and T770C were detected at the 3rd time point; the former occurred in the hydrophilic region of HBsAg and the latter was previously discovered in a HCC patient (Genbank number AY206393). We speculated that these mutants might afford HBV variants a distinct survival advantage by allowing the mutant virus to escape from the immune system. In addition, these mutations could further transactivate certain oncogenes which in turn rendered the acceleration of the liver damage [9–13].

### 4.2. An 18 bp Deletion of PreS1

In this patient, we found an 18 bp deletion of preS1 that was kept in. The deletion region is in the span of hepatocellular-binding site (aa5–20) and led to aa1–11 deletion in preS1. This deletion mutant was previously reported in different clinical cases and genotypes [14–17]. For example, this 18 bp deletion was detected in a heart transplant patient while it was not found with the donor [18]. In our case, retrospective study showed that the son of this patient, though infected with HBV via mother-to-infant transmission, carried none of these mutant strains in the sequenced clones [19]. This phenomenon may imply that host immune pressure was the primary cause of aa1–11 deletion in preS1. In addition, this mutant seemed to have reduced from the 1st time point to the 3rd time point (7/9 versus 7/9 versus 2/8) and had a tendency to be substituted by large fragments deletion located in the preS region at the 3rd time point (Figure 2). Whether it was a precursor of large fragment deletion mutation or just an isolated event happened under the immune pressure still needed to be carefully studied.

### 4.3. Defective Genomes during the HBV Evolution

**Figure 3:**
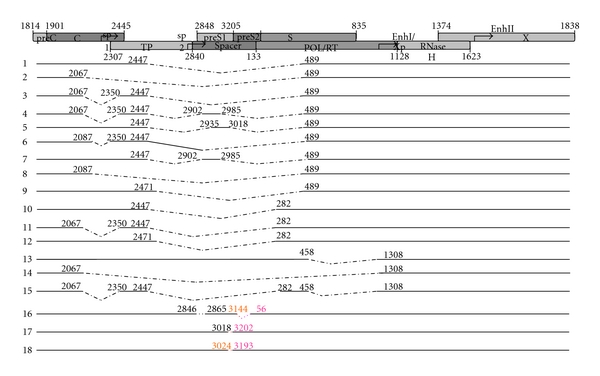
HBV spliced variants. There were 15 types of splicer reported in the literature, including seven 5′-splice sites at nucleotide positions 2447, 2067, 2985, 3018, 2087, 2471, 458; six 3′-splice sites at nucleotide positions 489, 2350, 2902, 2935, 282, 1308. In this report, 3 new splicers were detected, including two new 5′–splice donors (nt3144 and 3024) and three new 3′-splice acceptors (nt56, 3193, and 3202).

The other prominent variation at the 3rd time point was the defective genomes caused by the large fragment deletion focused on the overlapping regions of polymerase/preS1 and preS1/preS2. The length of internal deletions was from 126 bp to 1256 bp. Up until now, there have been at least 15 types of spliced HBV DNA reported in the literature (Figure 3) [20, 21], which consisted of seven 5′-splice donors and six 3′-splice accepters. In our case, four types of spliced HBV DNA were found at the 3rd time point, the donor site and the receptor site of these splicers followed the GT-AT rules [22]. Among them, the 2447/489 donor/acceptor splicer existed as described in previous reports and produced a presumptive HBV spliced protein (HBSP) [23]. In addition, positions 3144 and 3024 as the 5′-donor sites and positions 3202, 3193, and 56 as the 3′-acceptor sites were first reported in our study. During a subsequence screening, we also detected defective genomes in patients with hepatocirrhosis and CHB (GenBank accesson numbers: EU439017, EU306668, EU306669, EU330986, EU306676, EU306670). Though these defective genomes were different in structure, they all shared sequences from nucleotides position 489 to position 2447. This region contains all known signals required for conventional replication (DR1 and DR2), synthesis, and processing of pregenomic/C-mRNA, preC mRNA, and X mRNA, and pregenomic RNA encapsidation. In contrast, all lacked the complete pre-S1 and, or pre-S2 ORFs. Many surveys noted that spliced HBV DNA and pre-S deletion were more frequently detected in patients with severe liver disease [24–26] and often existed during the process of anti-HBe seroconversion and subsequent loss of HBsAg [27–29]. However, in this case with our subsequent screening, all the patients with defective genomes were HBeAg positive and with high HBV DNA load (≥10^7^ copy/mL). Based on these findings, we deduced that these defective genomes (with intact X gene and all signals required for viral life cycle) might contribute to persistent HBV replication in the patient.

### 4.4. Mutations Scattered in the Whole Genomes

Other mutations such as C934A, C2351T/A2353T, C2444T, A1762T/G1764A, and G1896A were scattered in the whole genomes. In the present study, all clones of the mother from three time points had double mutations of nucleotide A1762T/G1764A in basal core promoter (BCP), four of which were also coupled with G1896A. However, we did not observe any HBeAg/anti-HBeAg seroconversion at the three time points, nor did we detect the impact of BCP mutation on HBV DNA load [30, 31]. In addition, multisequences alignment has showed that genotype C had a tendency for higher prevalence of the BCP mutation (41 versus 35, 53.9%) (GenBank accesson number can be obtained on request). Combined with the clinic results (Table 1), whether the BCP and precore mutations in this patient were related to infection with genotype C, or whether the patient was seroconverting to anti-HBeAg or may develop severe disease [32, 33] are questions that still need to be followedup.

In conclusion, our data showed that viral quasispecies are very closely related to genomes but exist in an environment of mutation, selection, and competition, and thus lead to a dynamic and changing population. During this progress, some mutations were kept in and became the dominant strains, some new mutations emerged, and these strains may play important roles in the persistence of infection or the progression of disease.

## Figures and Tables

**Table 1:** Clinic data and HBV DNA load in serum samples.

	May 2005	Oct. 2005	Dec. 2007
HBsAg (OD/Cut-Off)	24.676	23.886	29.495
HBsAb (OD/Cut-Off)	0.048	0.010	0.248
HBeAg (OD/Cut-Off)	3.467	22.819	9.705
HBeAb (OD/Cut-Off)	1.869	1.785	2.385
HBcAb (OD/Cut-Off)	0	0.002	0
HBV-DNA (copy/mL^−1^)	*	2.4 × 10^8^	2.5 × 10^7^
ALT (U/L) (8–40) ^a^	27	51↑	105↑
AST (U/L) (5–40) ^a^	26	33	47↑
T-BIL (*μ*M) (3.4–20.5) ^a^	12	17.2	10.6
D-BIL (*μ*M) (0–6.8) ^a^	3	4.9	3.5
I-BIL (*μ*M) (0–13.7) ^a^	9	12.3	7.1
TP (g/L) (65–80) ^a^	73	71	70
ALB (g/L) (35–55) ^a^	33	44	45
GLO (g/L) (20–30) ^a^	40↑	27	25

*lack of serum.

^a^refernce values.

↑higher than reference values.
